# Two new wood-decaying fungal species on *Arundo donax* from Guangxi, southern China

**DOI:** 10.3897/mycokeys.134.194492

**Published:** 2026-06-10

**Authors:** Qian-Xin Guan, Heng Zhao, Li Wang, Chun-Ying Deng, Yu-Cheng Dai, Guang-Yu Zeng

**Affiliations:** 1 School of Ecology and Nature Conservation, Beijing Forestry University, Beijing 100083, China College of Economics and Management, Tianjin University of Science and Technology Tianjin China; 2 CAS Key Laboratory of Forest Ecology and Silviculture, Institute of Applied Ecology, Chinese Academy of Sciences, Shenyang 110164, China Institute of Applied Ecology, Chinese Academy of Sciences Shenyang China; 3 College of Economics and Management, Tianjin University of Science and Technology, Tianjin 300222, China Guizhou Institute of Biology, Guizhou Academy of Sciences Guiyang China; 4 Guizhou Institute of Biology, Guizhou Academy of Sciences, Guiyang, 550009, China School of Ecology and Nature Conservation, Beijing Forestry University Beijing China; 5 Guizhou Provincial Key Laboratory of Agricultural Microbiology, Guizhou Academy of Sciences, Guiyang, 550009, China Guizhou Provincial Key Laboratory of Agricultural Microbiology, Guizhou Academy of Sciences Guiyang China; 6 Guangxi Forestry Research Institute, Nanning 530002, Guangxi, China Guangxi Forestry Research Institute Guangxi China

**Keywords:** Agaricales, new taxon, Polyporales, species diversity, wood-rotted fungi

## Abstract

Wood-decaying fungi are among the most important groups of macrofungi with crucial ecological roles and economic value. In this study, phylogenies were reconstructed using ITS + nLSU genetic loci of *Phlebiopsis*, and ITS + nLSU + *tef1* genetic loci of *Sicyoideibasidia*. Two new wood-decaying fungal species on *Arundo donax*, *Phlebiopsis arundinacea***sp. nov**. and *Sicyoideibasidia luteocystidia***sp. nov**. are illustrated and described from Guangxi, southern China. *Phlebiopsis arundinacea* is characterized by resupinate, membranaceous basidiomata with smooth and grayish brown hymenial surface when fresh, a pseudodimitic hyphal system with simple-septate generative hyphae, the presence of brown skeletocystidia and lamprocystidia, and subcylindrical to oblong-ellipsoid basidiospores. *Sicyoideibasidia luteocystidia* is characterized by resupinate, membranaceous basidiomata with smooth and white to cream hymenial surface when fresh, a monomitic hyphal system bearing clamp connections on generative hyphae, the presence of fusiform to subulate and slight yellow clavate to capitate cystidia, and ellipsoid, thick-walled basidiospores. The phylogenetic analyses showed that *P. arundinacea* was closely related to *P. crassa*, with strong support based on the ITS + nLSU genetic loci. *Scopuloides hainanensis* was sister to *S. yunnanensis* and was grouped with other species of *Sicyoideibasidia*, both relationships strongly supported by ITS + nLSU + *tef1* genetic loci. A full description, illustrations, and phylogenetic analysis results of the two new species are provided here. In addition, *Sicyoideibasidia yunnanensis* is secondly recorded after its original description, and it was found on *Arundo donax* from Guangxi Autonomous Region, Southern China.

## Introduction

Fungi, the second largest group of organisms on Earth, inhabit an extraordinarily wide range of environments, making them one of the most diverse groups and playing essential roles in maintaining ecosystem stability (Schimann et al. 2017; Hyde et al. 2024; Zhao et al. 2025a). Wood-decaying fungi, in particular, play a particularly prominent role, either parasitizing or saprotrophically colonizing lignocellulosic substrates and exhibiting highly diverse hymenophore morphologies, such as poroid, corticioid, and hydnoid (Dai 2010; Dai et al. 2021; Ryvarden et al. 2022; Wang et al. 2023; Yuan et al. 2026; Zhao et al. 2026). Recently, the diversity of the Chinese macrofungi has been extensively studied and a large number of new taxa to China have been discovered, among which wood-decaying fungi are an important component (Wu et al. 2022a; Zhang et al. 2023; Dong et al. 2024, 2025a, 2025b; Liu et al. 2025; Wang et al. 2025; Wijesinghe et al. 2025; Yang et al. 2025; Zhao et al. 2025a, 2025b; Deng et al. 2026; Li et al. 2026; Luo et al. 2026; Wang et al. 2026; Zhuang et al. 2026; Zeng et al. 2026a). A large part of these new taxa is concentrated in the orders Agaricales and Polyporales, greatly enriching the species diversity of both orders (Guan et al. 2021; Ryvarden et al. 2022; Wu et al. 2022b; He et al. 2024; Zhao et al. 2024). Despite significant advances in species diversity among wood-inhabiting fungi of Agaricales and Polyporales in China (Cui et al. 2019; Shen et al. 2019; Dong et al. 2025a; Wang et al. 2025; Luo et al. 2026; Wang et al. 2026), systematic research on the species diversity of wood-inhabiting fungi is still required. In addition, many studies focused on the wood-decaying fungi growing on gymnosperms and angiosperms, and a few contracted on the monocotyledon, such as *Arundo donax* with a wide distribution range.

The genus *Phlebiopsis* Jülich was proposed with the type species *P. gigantea* (Fr.) Jülich, which belonged to Phanerochaetaceae, Polyporales, Agaricomycetes, and Basidiomycota (Floudas and Hibbett 2015; Zhao et al. 2021). The genus is characterized by resupinate to effused-reflexed, ceraceous, membranaceous to coriaceous basidiomata when fresh, smooth, tuberculate, odontoid, hydnoid to poroid hymenophore, a monomitic or dimitic hyphal structure with simple-septate generative hyphae, presence of encrusted cystidia, clavate basidia and basidioles, and hyaline, thin-walled, smooth, cylindrical to ellipsoid basidiospores which are negative in Cotton Blue Melzer’s reagent (Jülich 1978; Bernicchia and Gorjón 2010; Zhao et al. 2021). Recent multigene phylogenetic studies have revealed that the genus *Phlebiopsis* is one of the substantially supported lineages within the family Phanerochaetaceae, and numerous new species have been discovered and their taxonomic status has been elucidated, and a total of 36 species of *Phlebiopsis* is accepted (Floudas and Hibbett 2015; Justo et al. 2017; Zhao et al. 2019; Lima et al. 2020; Xu et al. 2020; Chen et al. 2021; Li et al. 2021; Zhao et al. 2020, 2021; Li et al. 2023; Dong et al. 2024; Deng et al. 2024; Manawasinghe et al. 2025; Motato-Vásquez et al. 2025).

The genus *Sicyoideibasidia* J.H. Dong & C.L. Zhao, typified by *S. bambusicola* J.H. Dong & C.L. Zhao, was proposed to belong to Cyphellopsidaceae, Agaricales, Agaricomycetes, and Basidiomycota (Dong et al. 2025a). Currently, a total of three species of *Sicyoideibasidia* is accepted, and these species are all distributed in Yunnan Province, China (Deng et al. 2025; Dong et al. 2025a). Morphologically, *Sicyoideibasidia* is characterized by annual, resupinate, adnate, membranaceous basidiomata, a monomitic hyphal system with clamp connections; presence of cystidia and gourd-shaped, 4 sterigmata basidia; and cylindrical, colorless, thin- to thick-walled, smooth basidiospores (Deng et al. 2025; Dong et al. 2025a).

Guangxi Autonomous Region, Southern China, has a subtropical monsoon climate with abundant sunshine and precipitation. Its rich woody plant resources support a high diversity of wood-decaying fungi, recording 956 species representing 2 phyla, 5 classes, 21 orders, 97 families, and 337 genera, and the phylum Basidiomycota (Zeng et al. 2026b). In this study, we describe two new species and one recorded species of corticioid wood-decaying fungi growing on *Arundo donax* from Guangxi, Southern China, based on morphological and phylogenetic analyses.

## Materials and methods

### Morphological studies

During a sampling trip to the Guangxi, China, in March 2026, seven corticioid wood-decaying fungi specimens were collected from dead culm of *Arundo donax*. Among these, two specimens were identified as a novel species of *Phlebiopsis*, and four specimens were identified as a novel species and a recorded species of *Sicyoideibasidia*, based on phylogeny and morphology. The voucher specimens were deposited in the herbarium of the Institute of Microbiology, Beijing Forestry University (**BJFC**), Beijing, China. The morphological descriptions are based on field notes and voucher specimens, following the methods outlined in previous studies (Mao et al. 2023; Liu et al. 2025; Zhang et al. 2025). For micro-morphological data, a light microscope was used to examine dried specimens. Sections were observed at magnifications up to 1000× using a Nikon Eclipse 80i microscope with phase contrast illumination (Nikon, Tokyo, Japan). Measurements, descriptions, and illustrations of microscopic features were derived from preparations mounted in Cotton Blue, KOH (5 %), Phloxine B (2 % C_20_H_4_Br_4_Cl_2_K_2_O_5_), and Melzer’s reagent (Acharya et al. 2017a, 2017b; Tarafder et al. 2018, 2023, 2025). To account for natural variability, the upper and lower 5% of extreme values were excluded from each dataset, with the adjusted ranges presented in parentheses (Dong et al. 2025). The following abbreviations are used: KOH = 5% potassium hydroxide; IKI = Melzer’s reagent; IKI– = neither amyloid nor dextrinoid; CB = Cotton Blue; CB– = acyanophilous; CB+ = cyanophilous; L = mean spore length; W = mean spore width; Q = length-to-width ratio; (n = x/y), where x is the number of spores measured and y is the number of specimens examined. Color descriptions follow Anonymous (1969) and Petersen (1996).

### DNA extraction, PCR, and sequencing

DNA was extracted from dried voucher specimens using a CTAB rapid kit (DN14, Aidlab Biotechnologies Co., Ltd., Beijing). The 25 μL polymerase chain reaction (PCR), including12.5 μL of PCR mix, 9.5 μL of ddH2O, 1 μL of forward primers, 1 μL of reverse primers, and 1 μL of DNA template, were performed according to the manufacturer’s instructions. The internal transcribed spacer (ITS), large subunit nuclear ribosomal RNA gene (nLSU), and translation elongation factor 1-α gene (*tef1*) partial regions were amplified using the ITS5/ITS4 (White et al. 1990), LROR/LR7 (https://sites.duke.edu/vilgalyslab/rdna_primers_for_fungi/), and 985F/1567R primers (Hopple and Vilgalys 1999), respectively.

The PCR procedure for ITS, nLSU, and *tef1* was as follows: initial denaturation at 95 °C for 3 min, followed by 34 cycles at 94 °C for 40 s, annealing at 54 °C for ITS and nLSU, and 56 °C for *tef1* for 45 s, and extension at 72 °C for 1 min, with a final extension at 72 °C for 10 min. The PCR products were purified and sequenced at the Beijing Genomics Institute (BGI), China, with the same primers as used in PCR. Newly generated sequences were deposited in GenBank. All sequences analyzed in this study are listed in Suppl. material 1.

### Phylogenetic analyses

In the present study, the phylogenetic relationships of *Phlebiopsis* were reconstructed using the concatenated dataset (ITS + nLSU) from voucher specimens, which included 52 specimens of *Phlebiopsis* and two specimens of *Rhizochaete* Gresl., Nakasone & Rajchenb. as the outgroups (Li et al. 2023). The phylogeny of *Sicyoideibasidia* was constructed based on dataset composed of concatenated ITS + nLSU + *tef1* sequences, and the members of *Nia* R.T. Moore & Meyers were selected as outgroups (Dong et al. 2025a). The ITS and nLSU partial sequences were separately aligned using MAFFT v.7 (Katoh and Standley 2013) and then concatenated into a combined dataset using PhyloSuite v1.2.3 (Xiang et al. 2023). The best partitioning schemes and evolutionary models were selected using PartitionFinder 2 v2.1.1 (Lanfear et al. 2017), with all algorithms and the AIC criterion, for two sets of pre-defined partitions: two partitions of *Phlebiopsis* (ITS and nLSU) and three partitions of *Sicyoideibasidia* (ITS, nLSU, and *tef1*).

Maximum Likelihood phylogenies were inferred using IQ-TREE (Nguyen et al. 2015) under Edge-linked partition model for 5,000 ultrafast (Minh et al. 2013) bootstraps, and Bayesian Inference phylogenies were inferred using MrBayes v3.2.7a (Ronquist et al. 2012) under partition model (2 parallel runs, two million generations of *Phlebiopsis*, and one million generations of *Sicyoideibasidia*), in which the initial 25% of sampled data were discarded as burn-in. The ML and BI trees were visualized using FigTree v1.4.3 (http://tree.bio.ed.ac.uk/software/figtree/), and nodes with ultrafast bootstrap support values ≥ 70 % and Bayesian Posterior Probabilities (BPP) ≥ 0.90 were marked on the ML tree.

## Results

### Phylogeny

**Figure 1.**
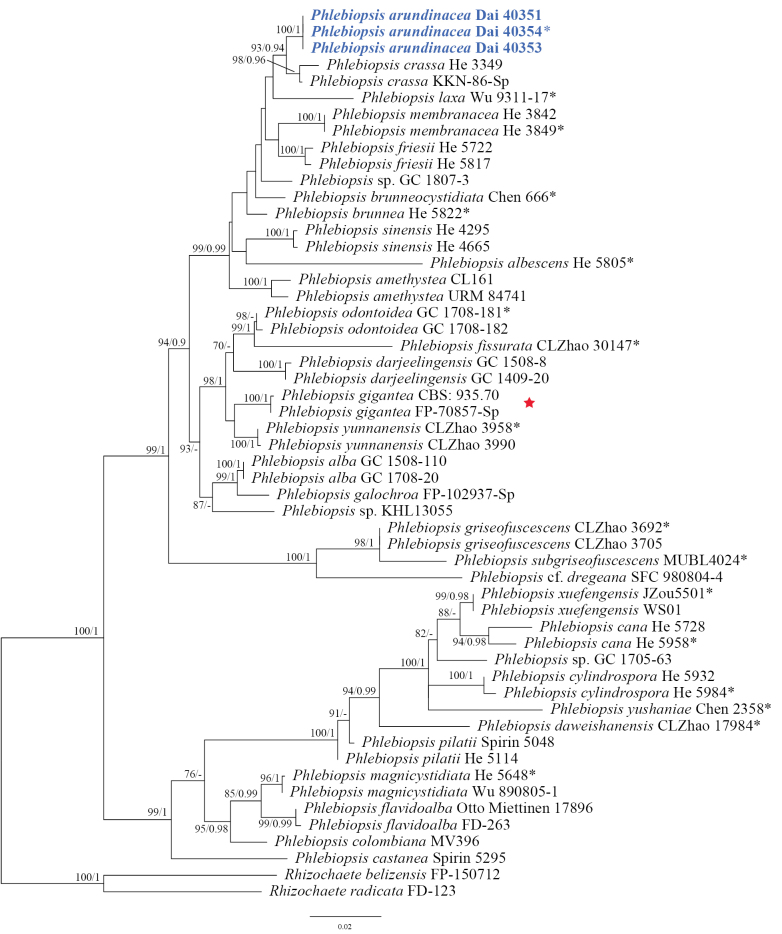
The ML and BI tree of *Phlebiopsis* were conducted based on a combined ITS + nLSU sequences. Branches are labeled with ultrafast bootstrap support values ≥ 70 % and Bayesian Posterior Probabilities (BPP) ≥ 0.90. The new species is in bold and blue; “*” represents to the type specimen; “star” represents to the type species.

The concatenated ITS + nLSU dataset of *Phlebiopsis* included sequences from 54 samples representing 31 taxa, and the dataset had an aligned length of 1,949 characters (1–617 characters for ITS, 618–1949 characters for nLSU). The best model applied for each region of the four partitions was ITS (TVM+I+G) and nLSU (GTR+I+G). Both ML and BI analyses yielded similar topologies (Fig. 1), with the ML tree selected for display, showing ultrafast bootstrap support values ≥ 70% and BPP ≥ 0.90. The BI analysis produced an average standard deviation of split frequencies of 0.007654. Phylogenetic analyses suggested that the three specimens, *P. arundinacea*, were closely related to *P. crassa* (Lév.) Floudas & Hibbett with well-supported values (93/0.94). Additionally, the *Phlebiopsis* species clustered into two groups, and all accepted *Phlebiopsis* species formed a distinct clade with high support. In addition, the BLAST comparison results of ITS sequences showed that the similarity between the new species and known species of *Phlebiopsis* was less than 98.5%. The BLAST comparison of ITS and nLSU sequences of three specimens of the new species is provided in Suppl. material 2.

**Figure 2.**
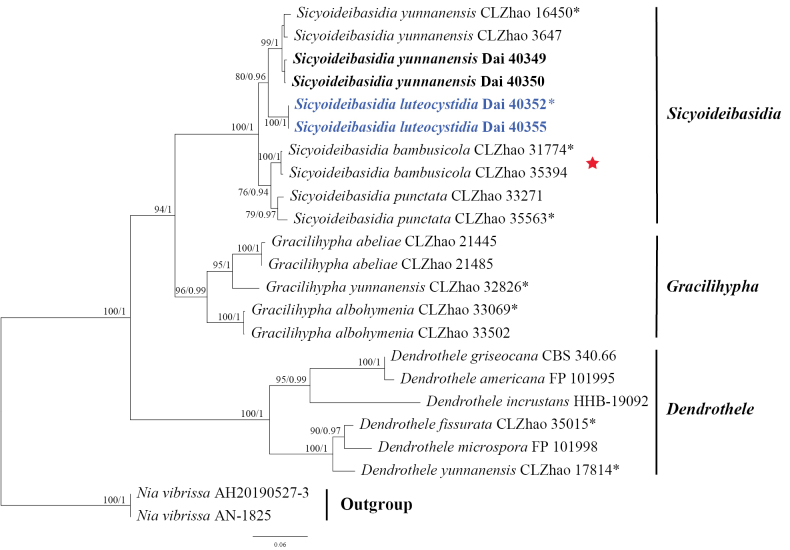
The ML and BI tree of *Sicyoideibasidia* and related genera were conducted based on a combined ITS + nLSU + *tef1* sequences. Branches are labeled with ultrafast bootstrap support values ≥ 70 % and Bayesian Posterior Probabilities (BPP) ≥ 0.90. The new species is in bold and blue; “*” represents to the type specimen. “star ” represents to the type species.

The concatenated ITS + nLSU + *tef1* dataset of *Sicyoideibasidia* included sequences from 23 samples representing 14 taxa, and the dataset had an aligned length of 3,181 characters (1–826 characters for ITS, 827–2546 characters for nLSU, and 2547–3181 characters for *tef1*). The best model applied for each region of the five partitions was ITS (TRN+I+G), nLSU (GTR+I+G), and *tef1* (TRNEF+G). Both ML and BI analyses yielded similar topologies (Fig. 2), with the ML tree selected for display, showing ultrafast bootstrap support values ≥ 70% and BPP ≥ 0.90. The BI analysis produced an average standard deviation of split frequencies of 0.002600. The phylogeny (Fig. 2) indicated the taxonomic relationship of *Sicyoideibasidia* and related genera. Our specimens formed two lineages within the *Sicyoideibasidia*. One of these lineages was distantly related to other taxa of *Sicyoideibasidia* and was described as a new species, *S. luteocystidia*. The other lineage contained two our specimens, which were identified as the known species *S. yunnanensis* Y.L. Deng & C.L. Zhao. The new species was sister to *S. yunnanensis* with well-supported values (80/0.96), and group with other species of *Sicyoideibasidia* with strong support (100/1). In addition, the BLAST comparison results of ITS sequences showed that the similarity between the new species and known species of *Sicyoideibasidia* was less than 95.5%. The BLAST comparison of ITS, nLSU, and *tef1* sequences of our four specimens of the *S. luteocystidia* and *S. yunnanensis* is provided in Suppl. material 2.

### Taxonomy

#### Phlebiopsis arundinacea

Taxon classificationFungiPolyporalesPhanerochaetaceae

Q.X. Guan, H. Zhao & Y.C. Dai sp. nov.

4E468801-90A9-5C14-91D5-1DFB2D8977F7

863901

Figs 3a, b, 4, 5

##### Holotype.

China • Guangxi Autonomous Region, Baise, Longlin County, Road 246, on dead culm of *Arundo donax*, elev. 744 m, 24.945080°N, 105.120492°E, 6 March 2026, Dai 40354 (BJFC065174).

**Figure 3.**
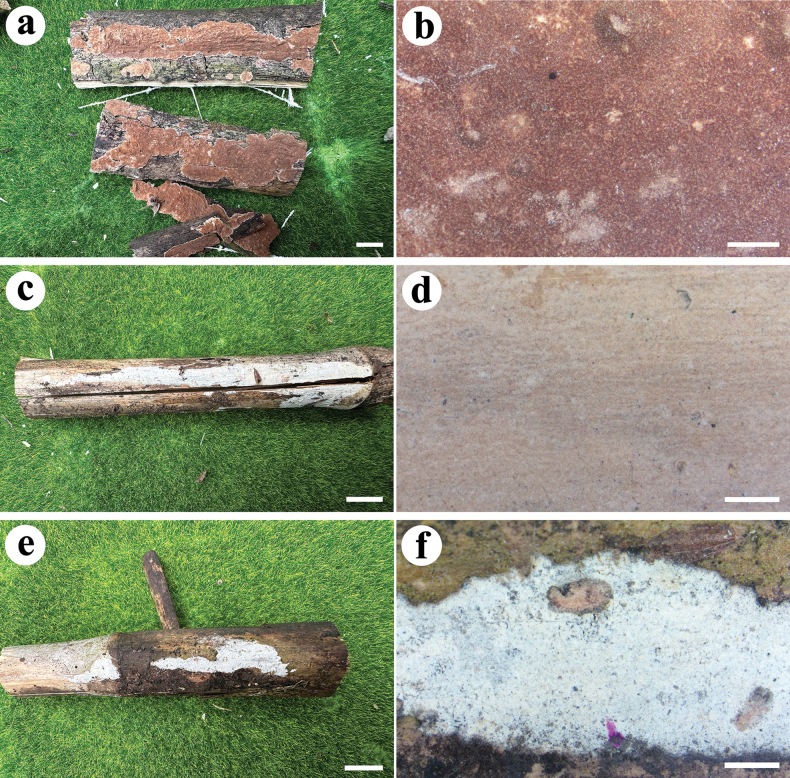
Basidiomata of *Phlebiopsis* and *Sicyoideibasidia*. **a, b**. *Phlebiopsis arundinacea* (holotype, Dai 40354); **c, d**. *Sicyoideibasidia luteocystidia* (holotype, Dai 40352); **e, f**. *Sicyoideibasidia yunnanensis*. Scale bars: 1 cm (**a, c, e**); 1 mm (**b, d, f**).

##### Etymology.

*Arundinacea* (Lat.) refers to the species growing on *Arundo donax*.

##### Basidiomata.

Annual, resupinate, adnate, membranaceous, without odor and taste when fresh, inseparable from the substrate, up to 10 cm long, 3 cm wide, 0.5 mm thick at center. Hymenial surface smooth, grayish brown when fresh, grayish brown to brown upon drying. Margin gradually thinning out, distinctly, cream to buff, up to 1 mm.

**Figure 4.**
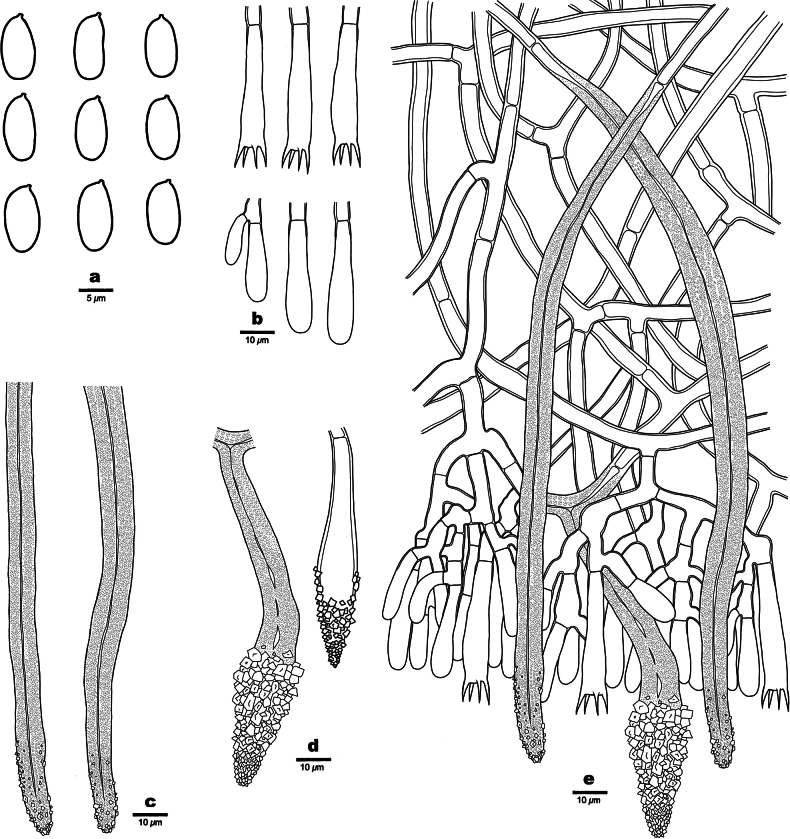
Microscopic structures of *Phlebiopsis arundinacea* (holotype, Dai 40354). **a**. Basidiospores; **b**. Basidia and basidioles; **c**. Skeletocystidia; **d**. Lamprocystidia; **e**. Part of the vertical section of basidiomata. Scale bars: 5 μm (**a**); 10 μm (**b–e**).

##### Hyphal structure.

Hyphal system pseudodimitic, generative hyphae simple-septate, colorless, thick-walled, branched, interwoven, 3.5–6.5 µm in diameter, IKI–, CB–; tissues unchanged in KOH. Skeletocystidia (skeletal hyphae) brown, distinctly thick-walled, apically encrusted with crystals, up to 240 µm long, 6–10 µm in diameter.

**Figure 5.**
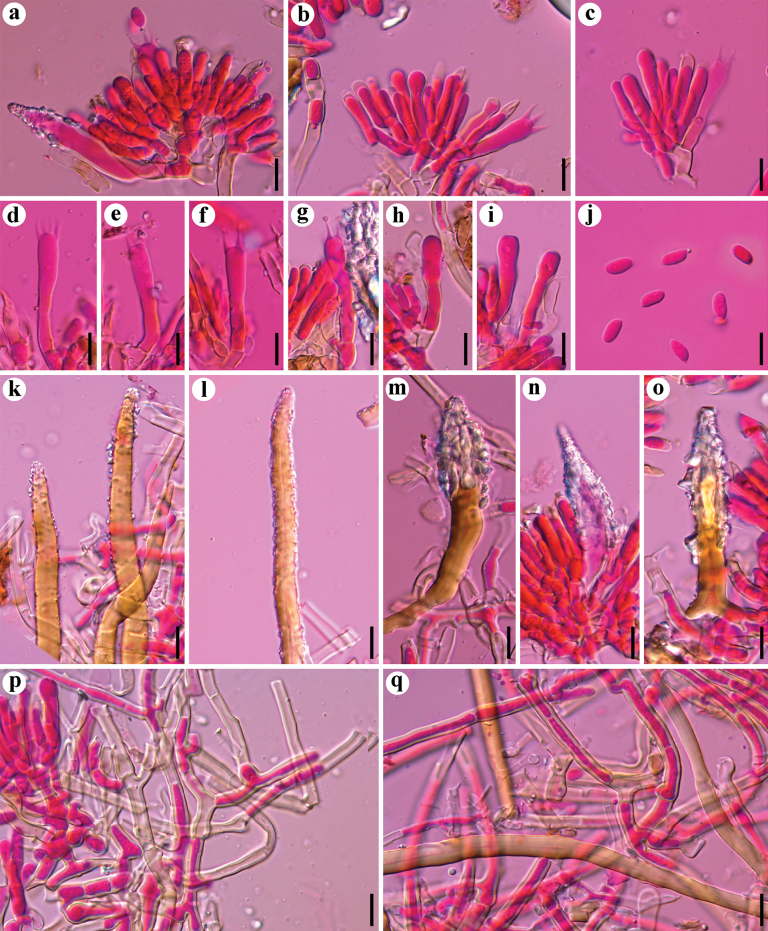
Sections of hymenia of *Phlebiopsis arundinacea* (holotype, Dai 40354). **a–i**. Basidia and basidioles; **j**. Basidiospores; **k, l**. Skeletocystidia; **m–o**. Lamprocystidia; **p, q**. Hyphae. Scale bars: 10 μm (**a–q**), 10 × 100 Oil.

##### Hymenium.

Lamprocystidia numerous, subulate to fusiform, usually brown, occasionally colorless, thick-walled, 50–100 × 10–18 µm. Basidia clavate, thin-walled, with 4 sterigmata and a basal simple septum, 32–38 × 6–8 µm; basidioles numerous, in shape similar to basidia but smaller. Basidiospores subcylindrical to oblong-ellipsoid, colorless, thin-walled, smooth, IKI–, CB–, (6.2–)6.3–9.4(–9.5) × 3.8–4.6 µm, L = 7.98 µm, W = 4.19 µm, Q = 1.69–2.11 (n = 60/2).

##### Type of rot.

White rot.

##### Additional specimens examined

**(paratypes)**. China • Guangxi Autonomous Region, Baise, Longlin County, Road 246, on dead culm of *Arundo donax*, elev. 744 m, 24.945080°N, 105.120492°E, 6 March 2026, Dai 40351 (BJFC065171), Dai 40353 (BJFC065173).

#### Sicyoideibasidia luteocystidia

Taxon classificationFungiPolyporalesPhanerochaetaceae

Q.X. Guan, H. Zhao & Y.C. Dai sp. nov.

10670830-1640-5375-8B83-AF089618CD9C

863903

Figs 3c, d, 6, 7

##### Holotype.

China • Guangxi Autonomous Region, Baise, Longlin County, Road 246, on dead culm of *Arundo donax*, elev. 744 m, 24.945080°N, 105.120492°E, 6 March 2026, Dai 40352 (BJFC065172).

##### Etymology.

*Luteocystidia* (Lat.) refers to the species having slightly yellow cystidia.

##### Basidiomata.

Annual, resupinate, adnate, membranaceous, without odor or taste when fresh, difficult to separate from substrate, up to 12 cm long, 1 cm wide, 0.1 mm thick. Hymenial surface smooth, white to cream when fresh, turning to cream upon drying. Margin gradually thinning out, concolorous with the hymenial.

**Figure 6.**
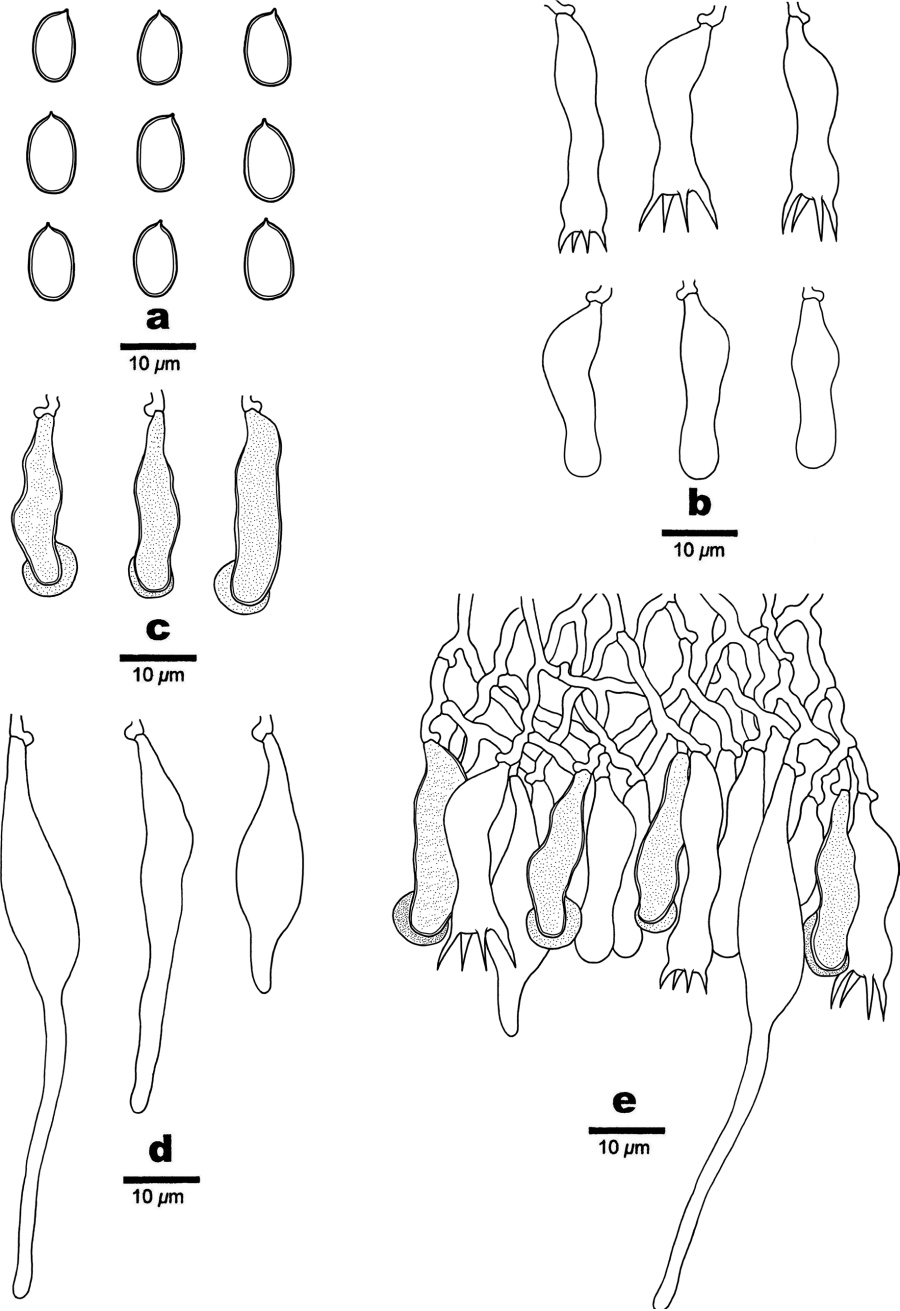
Microscopic structures of *Sicyoideibasidia luteocystidia* (holotype, Dai 40352). **a**. Basidiospores; **b**. Basidia and basidioles; **c**. Clavate to capitate cystidia; **d**. Fusiform to subulate cystidia; **e**. Part of the vertical section of hymenium. Scale bars: 10 μm (**a–e**).

##### Hyphal structure.

Hyphal system monomitic, generative hyphae with clamp connections, colorless, thin-walled, branched, interwoven, 1.5–2 μm in diameter, IKI–, CB–; tissues unchanged in KOH.

**Figure 7.**
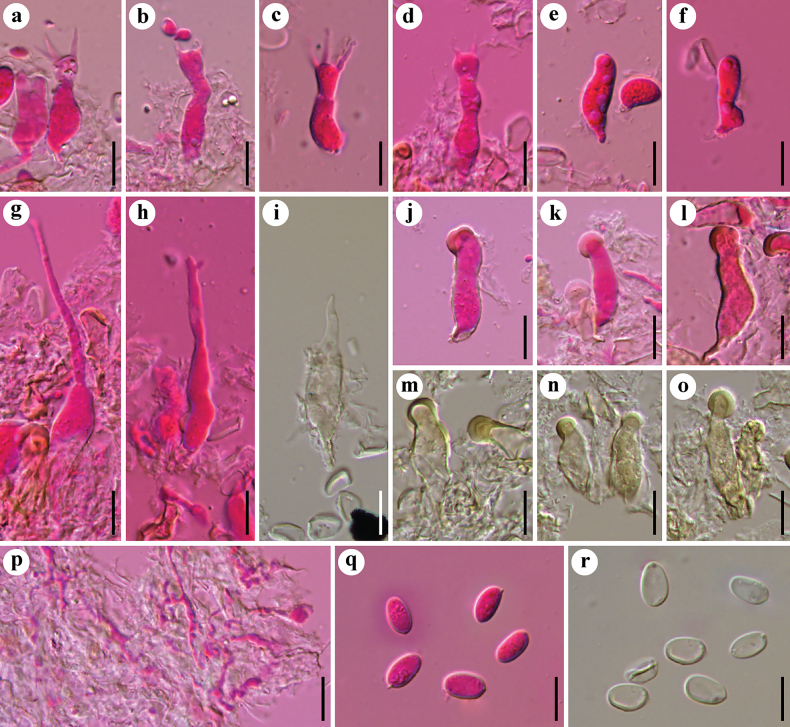
Sections of hymenium of *Sicyoideibasidia luteocystidia* (holotype, Dai 40352). **a–f**. Basidia and basidioles; **g–i**. Clavate to capitate cystidia; **j–o**. Fusiform to subulate cystidia; **p**. Hyphae; **q, r**. Basidiospores. Scale bars: 10 μm (**a–q**), 10 × 100 Oil.

##### Hymenium.

Cystidia of two types: (1) fusiform to subulate, colorless, thin-walled, 35–75 × 6–9 µm; (2) clavate to capitate, slight yellow, thick-walled, distinctly thickened at the apex, 18–30 × 5–8.5 µm. Basidia gourd-shaped, slightly constricted in the middle, with 2–4 sterigmata and a basal clamp connection, 20–30 × 5–8 µm; basidioles numerous, in shape similar to basidia but smaller. Basidiospores ellipsoid, colorless, thick-walled, smooth, IKI–, CB–, 8.8–10.5(–10.6) × 4.8–6.5 µm, L = 9.61 µm, W = 5.68 µm, Q = 1.67–1.71 (n = 60/2).

##### Type of rot.

White rot.

##### Additional specimen examined

**(paratype)**. China • Guangxi Autonomous Region, Baise, Longlin County, Road 246, on dead culm of *Arundo donax*, elev. 744 m, 24.945080°N, 105.120492°E, 6 March 2026, Dai 40355 (BJFC065175).

#### Sicyoideibasidia yunnanensis

Taxon classificationFungiPolyporalesPhanerochaetaceae

Y.L. Deng & C.L. Zhao, in Deng, Chen, Liu, Wang, Liu, Li & Zhao, Mycosystema 44(11, no. 250159): 7 (2025)

FFA12076-4BB5-54B4-9BC5-95F73C767ABE

859411

Fig. 3e, f

##### Description.

For a detailed description of *Sicyoideibasidia yunnanensis* see the Deng et al. (2025). This is secondly recorded of the species after its original description.

##### Material examined.

China • Guangxi Autonomous Region, Baise, Longlin County, Road 246, on dead culm of *Arundo donax*, elev. 744 m, 24.945080°N, 105.120492°E, 6 March 2026, Dai 40349 (BJFC065169), Dai 40350 (BJFC065170).

## Discussion

China, spanning a wide latitudinal and environmental gradient from boreal forests to tropical rainforests, provides an ideal natural framework for examining large-scale patterns of fungal diversity, especially in tropical and subtropical regions, and it is estimated to have high fungal diversity (Zhao et al. 2024; Bao et al. 2025; Zeng et al. 2026a). Guangxi Autonomous Region, located in southern China, features a typical subtropical monsoon climate with abundant sunlight and ample precipitation in which it is extremely rich in woody plant resources, providing excellent ecological conditions for the growth of wood-decaying fungi (Zeng et al. 2026b). The recent systematic investigations of macrofungi in the Guangxi region have identified nearly 1,000 species of wood-inhabiting fungi, mainly distributed in the orders Polyporales, Hymenochaetales, Agaricales, and Russulales (Zeng et al. 2026b). This indicates the region’s rich diversity of wood-inhabiting fungi and suggests that even more remains to be discovered. Recently, some new species have been discovered in Guangxi, growing on gymnosperms and angiosperms (Liu et al. 2023; Huang and Lin 2024; Liu et al. 2025; Shao et al. 2025). However, species that grow on Poaceae are not reported. In the present study, the discovery of *Phlebiopsis arundinacea* sp. nov., *Sicyoideibasidia luteocystidia* sp. nov. and known species *S. yunnanensis* confirmed that more taxa growing on *Arundo donax* (Poaceae) may be found after further sampling on this special substrate.

The new species *Phlebiopsis arundinacea* formed one distinct lineage with robust support, and it was closely related to *P. crassa* (Fig. 1). However, morphologically, *P. crassa* differs from *P. arundinacea* by effuse-reflexed basidiomata with purple tints in the hymenial surfaces, longer cystidia (80–150 × 6–12 µm vs. 50–100 × 10–18 µm; Burdsall 1985). The genus *Sicyoideibasidia* is closely related to *Gracilihypha* Yang Yang & C.L. Zhao (Fig. 2), consistent with previous study (Dong et al. 2025a). The new species *Sicyoideibasidia luteocystidia* formed one distinct lineage and is sister to *S. yunnanensis*, but *S. yunnanensis* differs from *S. luteocystidia* by having wider fusiform to subulate cystidia (41–65 × 11–13 µm vs. 35–75 × 6–9 µm) and larger basidiospores (12.8–16.3 × 6.8–10.1 µm vs. 8.8–10.5 × 4.8–6.5 µm; Deng et al. 2025).

Morphologically, the *Phlebiopsis* species can be divided into two groups, in which one group has poroid, irpicoid, or hydnoid hymenial surfaces, while the other has smooth, tuberculate or odontoid surfaces. The new species *P. arundinacea* may be confused with *P. bambusicola* (Berk. & Broome) Nakasone & S.H. He, *P. brunnea* Y. Nan Zhao & S.H. He, *P. novae-granatae* (A.L. Welden) Nakasone & S.H. He, and *P. sinensis* Y. Nan Zhao & S.H. He by sharing smooth, tuberculate, or odontoid, brown or slightly brown hymenial surface, and presence of skeletocystidia. However, *P. bambusicola* differs from *P. arundinacea* by its thinner basidiospores (6–7 × 2.5–3 µm vs. 6.3–9.4 × 3.8–4.6 µm; Hjortstam and Ryvarden 2005); *P. brunnea* is distinguished from *P. arundinacea* by its colorless lamprocystidia, and smaller basidia (20–33 × 4.5–6 µm vs. 32–38 × 6–8 µm; Zhao et al. 2021); *P. novae-granatae* differs from *P. arundinacea* by its monomitic hyphal system, lacking lamprocystidia, and grows on bamboo (Welden 1975; Hjortstam and Ryvarden 1990); *P. sinensis* is distinguished from *P. arundinacea* by its basidiomata with reflexed edges, smaller lamprocystidia (30–60 × 8–13 µm vs. 50–100 × 10–18 µm) and basidia (20–30 × 4.5–5.5 µm vs. 32–38 × 6–8 µm), and narrower basidiospores (5.8–7.8 × 2.5–3.5 µm vs. 6.3–9.4 × 3.8–4.6 µm; Zhao et al. 2021). In the genus *Sicyoideibasidia*, the new species *S. luteocystidia* may be confused with *S. bambusicola* and *S. punctata* by sharing resupinate, adnate, membranaceous basidiomata with white to cream hymenial surface, but differs in having clavate to capitate, slight yellow, thick-walled cystidia, smaller basidiospores (8.8–10.5 × 4.8–6.5 µm vs. 9.8–12.2 × 5.5–7 µm in *S. bambusicola* and 9–12 × 5.5– 6.8 µm in *S. punctata*; Dong et al. 2025a).

## Supplementary Material

XML Treatment for Phlebiopsis arundinacea

XML Treatment for Sicyoideibasidia luteocystidia

XML Treatment for Sicyoideibasidia yunnanensis

## Additional information

### Artificial Intelligence (AI) use

The authors accept full responsibility for the content of the manuscript, including the disclosure of any use of AI.

No AI tools were used in the preparation of this manuscript.

### Author ORCIDs

Q.-X. Guan https://orcid.org/0000-0002-7072-080X

H. Zhao https://orcid.org/0000-0003-2938-5613

L. Wang https://orcid.org/0009-0007-9331-6600

C.-Y. Deng https://orcid.org/0000-0002-0960-0948

Y.-C. Dai https://orcid.org/0000-0002-6523-0320

G.-Y. Zeng https://orcid.org/0009-0009-8609-1612

### Data availability

All of the data that support the findings of this study are available in the main text or Supplementary Information.

## Supplementary materials

Supplementary material 1 A list of species, specimens, and GenBank accession numbers of sequences used in this study Data type docx Explanation note “–” refers to the unavailability of the data. “*” represents to the type specimen. https://binary.pensoft.net/file/1675727This dataset is made available under the Open Database License (http://opendatacommons.org/licenses/odbl/1.0/). The Open Database License (ODbL) is a license agreement intended to allow users to freely share, modify, and use this Dataset while maintaining this same freedom for others, provided that the original source and author(s) are credited. Qian-Xin Guan, Heng Zhao, Li Wang, Chun-Ying Deng, Yu-Cheng Dai, Guang-Yu Zeng

Supplementary material 2 BLAST comparison of ITS, nLSU, and tef1 sequences for our specimens from the NCBI database https://doi.org/10.3897/mycokeys.134.194492.suppl2 Data type docx https://binary.pensoft.net/file/1675728This dataset is made available under the Open Database License (http://opendatacommons.org/licenses/odbl/1.0/). The Open Database License (ODbL) is a license agreement intended to allow users to freely share, modify, and use this Dataset while maintaining this same freedom for others, provided that the original source and author(s) are credited. Qian-Xin Guan, Heng Zhao, Li Wang, Chun-Ying Deng, Yu-Cheng Dai, Guang-Yu Zeng
