# Supplementary material for: Two new wood-decaying fungal species on Arundo donax from Guangxi, southern China
Source: MycoKeys. 2026 Jun 10;134:27–46. doi: 10.3897/mycokeys.134.194492 (PMC13276499; doi:10.3897/mycokeys.134.194492)
Supplement: Supplementary material 1 — A list of species, specimens, and GenBank accession numbers of sequences used in this study [file mycokeys-134-027-s001.docx]

**Supplementary Table 1.** A list of species, specimens, and GenBank accession numbers of sequences used in this study. “–” refers to the unavailability of the data. “*” represents to the type specimen.

| **Species** | **Specimen no.** | **Location** | **GenBank accession no.** | | |
| --- | --- | --- | --- | --- | --- |
|  |  |  | **ITS** | **nLSU** | ***tef1*** |
| *Dendrothele americana* | FP101995* | USA | – | NG071235 | – |
| *Dendrothele fissurata* | CLZhao 35015* | China | PV197921 | – | – |
| *Dendrothele griseocana* | CBS 340.66 | France | MH858816 | MH870455 | – |
| *Dendrothele incrustans* | HHB-19092 | – | MW740330 | – | – |
| *Dendrothele microspora* | FP 101998 | – | OQ694474 | OQ694474 | – |
| *Dendrothele yunnanensis* | CLZhao 17814* | China | OR094484 | OR449910 | – |
| *Gracilihypha abeliae* | CLZhao 21445 | China | PP819705 | PP826257 | – |
| *Gracilihypha abeliae* | CLZhao 21485 | China | PP819706 | PP826258 | – |
| *Gracilihypha albohymenia* | CLZhao 33069* | China | PV197927 | – | – |
| *Gracilihypha albohymenia* | CLZhao 33502 | China | PV197928 | – | PV231421 |
| *Gracilihypha yunnanensis* | CLZhao 32826* | China | PV197929 | PV197943 | – |
| *Nia vibrissa* | AH20190527-3 | Japan | LC769783 | LC769838 | – |
| *Nia vibrissa* | AN-1825 | Japan | LC769781 | LC769836 | – |
| *Phlebiopsis alba* | GC 1508-110 | China | MZ637042 | MZ637246 | – |
| *Phlebiopsis alba* | GC 1708-20 | China | MZ637043 | MZ637247 | – |
| *Phlebiopsis albescens* | He 5805* | China | MT452526 |  | – |
| *Phlebiopsis amethystea* | CL161 | Brazil | MK993644 | MK993638 | – |
| *Phlebiopsis amethystea* | URM84741 | China | MK993645 | MK993639 | – |
| ***Phlebiopsis arundica*** | **Dai 40351** | **China** | **PZ253611** | **PZ253604** | – |
| ***Phlebiopsis arundica*** | **Dai 40353** | **China** | **PZ253612** | **PZ253605** | – |
| ***Phlebiopsis arundica*** | **Dai 40354*** | **China** | **PZ253613** | **PZ253606** | – |
| *Phlebiopsis brunnea* | He 5822* | China | MT452527 | MT447451 | – |
| *Phlebiopsis brunneocystidiata* | Chen 666* | China | MT561707 | GQ470640 | – |
| *Phlebiopsis cana* | He 5728 | Sri Lanka | – | ON963991 | – |
| *Phlebiopsis cana* | He 5958∗ | China | – | ON963992 | – |
| *Phlebiopsis castanea* | Viacheslav Spirin 5295 | Russia | KX752610 | KX752610 | – |
| *Phlebiopsis cf. dregeana* | SFC 980804-4 | Korea | AF479669 | – | – |
| *Phlebiopsis colombiana* | MV396 | – | PV562816 | PV562976 | – |
| *Phlebiopsis crassa* | He 3349 | China | MT561712 | MT447407 | – |
| *Phlebiopsis crassa* | KKN-86 | USA | KP135394 | KP135215 | – |
| *Phlebiopsis cylindrospora* | He 5932 | China | MT386403 | MT447444 | – |
| *Phlebiopsis cylindrospora* | He 5984* | China | MT386404 | MT447445 | – |
| *Phlebiopsis darjeelingensis* | GC 1508-8 | China | MZ637053 | MZ637253 | – |
| *Phlebiopsis darjeelingensis* | GC 1409-20 | China | MZ637052 | MZ637252 | – |
| *Phlebiopsis daweishanensis* | CLZhao 17984* | China | OR096193 | OR461451 | – |
| *Phlebiopsis fissurata* | CLZhao 30147* | China | OR917877 | OR921223 | – |
| *Phlebiopsis flavidoalba* | Otto Miettinen 17896 | USA | KX752607 | KX752607 | – |
| *Phlebiopsis flavidoalba* | FD-263 | USA | KP135402 | KP135271 | – |
| *Phlebiopsis friesii* | He 5722 | Sri Lanka | MT452528 | MT447413 | – |
| *Phlebiopsis friesii* | He 5817 | Sri Lanka | MT452529 | MT447414 | – |
| *Phlebiopsis galochroa* | FP-102937-Sp | USA | KP135391 | KP135270 | – |
| *Phlebiopsis gigantea* | CBS: 935.70 | Germany | MH860011 | MH871798 | – |
| *Phlebiopsis gigantea* | FP-70857-Sp | USA | KP135390 | KP135272 | – |
| *Phlebiopsis griseofuscescens* | CLZhao 3692* | China | MT180946 | MT180950 | – |
| *Phlebiopsis griseofuscescens* | CLZhao 3705 | China | MT180947 | MT180951 | – |
| *Phlebiopsis laxa* | Wu 9311-17* | China | MT561710 | GQ470649 | – |
| *Phlebiopsis magnicystidiata* | He 5648* | China | MT386377 | MT447409 | – |
| *Phlebiopsis magnicystidiata* | Wu 890805-1 | China | MT561711 | GQ470667 | – |
| *Phlebiopsis membranacea* | He 3842 | China | MT386400 | – | – |
| *Phlebiopsis membranacea* | He 3849* | China | MT386401 | – | – |
| *Phlebiopsis odontoidea* | GC 1708-181* | China | MZ637054 | MZ637255 | – |
| *Phlebiopsis odontoidea* | GC 1708-182 | China | MZ637055 | MZ637256 | – |
| *Phlebiopsis pilatii* | He 5114 | China | MT386385 | – | – |
| *Phlebiopsis pilatii* | Viacheslav Spirin 5048 | Russia | KX752590 | KX752590 | – |
| *Phlebiopsis sinensis* | He 4295 | China | MT386395 | – | – |
| *Phlebiopsis sinensis* | He 4665 | China | MT386396 | – | – |
| *Phlebiopsis sp.* | GC 1705-63 | China | MZ637058 | MZ637258 | – |
| *Phlebiopsis sp.* | GC 1807-3 | USA | MZ637059 | MZ637259 | – |
| *Phlebiopsis sp.* | KHL13055 | Costa Rica | EU118662 | EU118662 | – |
| *Phlebiopsis subgriseofuscescens* | MUBL4024* | India | OP101632 | OP379562 | – |
| *Phlebiopsis xuefengensis* | JZou5501* | China | MT554931 | MT554924 | – |
| *Phlebiopsis xuefengensis* | WS01 | China | MT554921 | MT554928 | – |
| *Phlebiopsis yunnanensis* | CLZhao 3958* | China | MH744140 | MH744142 | – |
| *Phlebiopsis yunnanensis* | CLZhao 3990 | China | MH744141 | MH744143 | – |
| *Phlebiopsis yushaniae* | Chen 2358* | China | MZ637047 | MZ637261 | – |
| *Rhizochaete belizensis* | FP-150712 | Belize | KP135408 | KP135280 | – |
| *Rhizochaete radicata* | FD-123 | USA | KP135407 | KP135279 | – |
| *Sicyoideibasidia bambusicola* | CLZhao 31774 * | China | PV197922 | PV197940 | PV231417 |
| *Sicyoideibasidia bambusicola* | CLZhao 35394 | China | PV197923 | PV197941 | PV231418 |
| ***Sicyoideibasidia luteocystidia*** | **Dai 40352*** | **China** | **PZ253616** | **PZ253609** | **PZ246675** |
| ***Sicyoideibasidia luteocystidia*** | **Dai 40355** | **China** | **PZ253617** | **PZ253610** | **PZ246676** |
| *Sicyoideibasidia punctata* | CLZhao 35563* | China | PQ609291 | PV197942 | PV231419 |
| *Sicyoideibasidia punctata* | CLZhao 33271 | China | PQ609290 | – | – |
| ***Sicyoideibasidia yunnanensis*** | **Dai 40349** | **China** | **PZ253614** | **PZ253607** | **PZ246673** |
| ***Sicyoideibasidia yunnanensis*** | **Dai 40350** | **China** | **PZ253615** | **PZ253608** | **PZ246674** |
| *Sicyoideibasidia yunnanensis* | CLZhao 16450* | China | PV441147 | – | PV730093 |
| *Sicyoideibasidia yunnanensis* | CLZhao 3647 | China | PV441148 | PV441161 | PV730092 |
