# Supplementary material for: Two new wood-decaying fungal species on Arundo donax from Guangxi, southern China
Source: MycoKeys. 2026 Jun 10;134:27–46. doi: 10.3897/mycokeys.134.194492 (PMC13276499; doi:10.3897/mycokeys.134.194492)
Supplement: Supplementary material 2 — BLAST comparison of ITS, nLSU, and tef1 sequences for our specimens from the NCBI database [file mycokeys-134-027-s002.docx]

**Supplementary Table 2.** BLAST comparison of ITS, nLSU, and *tef1* sequences for our specimens from the NCBI database.

| **Locus** | **Rank** | **Matched Species** | **Voucher** | **Identity (%)** | **Max Score** | **Query Cov. (%)** | **Align. Length** | **Accession No.** |
| --- | --- | --- | --- | --- | --- | --- | --- | --- |
| ITS  Dai 40351 | 1 | *P. crassa* | 2-F9 | 98.06 | 811 | 100 | 663 | MW081305 |
|  | 2 | *P. crassa* | He 6304 | 98.06 | 811 | 100 | 641 | MT561714 |
|  | 3 | *P. gigantea* | 584 | 97.85 | 808 | 100 | 609 | PP844435 |
|  | 4 | *P. gigantea* | 11A | 97.85 | 808 | 100 | 596 | JQ764767 |
|  | 5 | *P. gigantea* | Wi-XII-2.1 | 97.85 | 808 | 100 | 677 | MF476018 |
| ITS  Dai 40353 | 1 | *P. crassa* | 2-F9 | 98.06 | 811 | 100 | 663 | MW081305 |
|  | 2 | *P. crassa* | He 6304 | 98.06 | 811 | 100 | 641 | MT561714 |
|  | 3 | *P. gigantea* | 584 | 97.85 | 808 | 100 | 609 | PP844435 |
|  | 4 | *P. gigantea* | 11A | 97.85 | 808 | 100 | 596 | JQ764767 |
|  | 5 | *P. gigantea* | Wi-XII-2.1 | 97.85 | 808 | 100 | 677 | MF476018 |
| ITS  Dai 40354 | 1 | *P. crassa* | 2-F9 | 98.06 | 811 | 100 | 663 | MW081305 |
|  | 2 | *P. crassa* | He 6304 | 98.06 | 811 | 100 | 641 | MT561714 |
|  | 3 | *P. gigantea* | 584 | 97.85 | 808 | 100 | 609 | PP844435 |
|  | 4 | *P. gigantea* | 11A | 97.85 | 808 | 100 | 596 | JQ764767 |
|  | 5 | *P. gigantea* | Wi-XII-2.1 | 97.85 | 808 | 100 | 677 | MF476018 |
| nLSU  Dai 40351 | 1 | *Phlebiopsis* sp. | He 5855 | 99.85 | 2481 | 100 | 1394 | MT447450 |
|  | 2 | *P. crassa* | He 6304 | 99.85 | 2481 | 100 | 1390 | MT598029 |
|  | 3 | *P. crassa* | He 5866 | 99.78 | 2475 | 100 | 1385 | MT447408 |
|  | 4 | *Phlebiopsis* sp. | He 5205 | 99.78 | 2475 | 100 | 1388 | MT447448 |
|  | 5 | *P. crassa* | MAFF 420737 | 99.78 | 2475 | 100 | 8049 | AB809163 |
| nLSU  Dai 40353 | 1 | *P. crassa* | He 6304 | 99.85 | 2490 | 100 | 1390 | MT598029 |
|  | 2 | *Phlebiopsis* sp. | He 5855 | 99.85 | 2490 | 100 | 1394 | MT447450 |
|  | 3 | *Phlebiopsis* sp. | He 5205 | 99.78 | 2475 | 100 | 1388 | MT447448 |
|  | 4 | *P. crassa* | MAFF 420737 | 99.78 | 2475 | 100 | 8049 | AB809163 |
|  | 5 | *P. sinensis* | He 4673 | 99.78 | 2484 | 100 | 1394 | MT447435 |
| nLSU  Dai 40354 | 1 | *Phlebiopsis* sp. | He 5855 | 99.85 | 2512 | 100 | 1394 | MT447450 |
|  | 2 | *P. crassa* | He 6304 | 99.78 | 2508 | 100 | 1390 | MT598029 |
|  | 3 | *P. crassa* | He 3349 | 99.78 | 2505 | 100 | 1391 | MT447407 |
|  | 4 | *P. sinensis* | He 4665 | 99.78 | 2505 | 100 | 1388 | MT447434 |
|  | 5 | *P. crassa* | MAFF 420737 | 99.71 | 2503 | 100 | 8049 | AB809163 |
| ITS  Dai 40349 | 1 | *S. yunnanensis* | CLZhao 11213 | 98.81 | 1199 | 100 | 740 | PV441146 |
|  | 2 | *S. yunnanensis* | CLZhao 3647 | 98.22 | 1177 | 100 | 687 | PV441148 |
|  | 3 | *S. yunnanensis* | CLZhao 16450 | 97.78 | 1162 | 100 | 727 | PV441147 |
|  | 4 | *S. bambusicola* | CLZhao 35394 | 95.52 | 1066 | 99 | 730 | PV197923 |
|  | 5 | *S. bambusicola* | CLZhao 31774 | 95.09 | 1053 | 99 | 736 | PV197922 |
| ITS  Dai 40350 | 1 | *S. yunnanensis* | CLZhao 11213 | 99.55 | 1277 | 100 | 740 | PV441146 |
|  | 2 | *S. yunnanensis* | CLZhao 3647 | 98.96 | 1205 | 100 | 687 | PV441148 |
|  | 3 | *S. yunnanensis* | CLZhao 16450 | 97.64 | 1157 | 100 | 727 | PV441147 |
|  | 4 | *S. bambusicola* | CLZhao 35394 | 94.92 | 1042 | 99 | 730 | PV197923 |
|  | 5 | *S. bambusicola* | CLZhao 31774 | 94.79 | 1040 | 99 | 736 | PV197922 |
| ITS  Dai 40352 | 1 | *S. yunnanensis* | CLZhao 11213 | 95.36 | 1061 | 100 | 740 | PV441146 |
|  | 2 | *S. yunnanensis* | CLZhao 16450 | 95.23 | 1057 | 100 | 727 | PV441147 |
|  | 3 | *S. yunnanensis* | CLZhao 3647 | 94.76 | 1037 | 100 | 687 | PV441148 |
|  | 4 | *S. bambusicola* | CLZhao 35394 | 94.62 | 1031 | 99 | 730 | PV197923 |
|  | 5 | *S. bambusicola* | CLZhao 31774 | 94.48 | 1027 | 99 | 736 | PV197922 |
| ITS  Dai 40355 | 1 | *S. yunnanensis* | CLZhao 11213 | 95.27 | 1103 | 100 | 740 | PV441146 |
|  | 2 | *S. yunnanensis* | CLZhao 16450 | 94.99 | 1088 | 100 | 727 | PV441147 |
|  | 3 | *S. yunnanensis* | CLZhao 3647 | 94.76 | 1066 | 100 | 687 | PV441148 |
|  | 4 | Agaricales sp. | He 5464 | 93.98 | 1050 | 100 | 719 | PQ842625 |
|  | 5 | *S. bambusicola* | CLZhao 31774 | 94.36 | 1029 | 96 | 736 | PV197922 |
| nLSU  Dai 40349 | 1 | *S. yunnanensis* | CLZhao 11213 | 99.74 | 1382 | 100 | 856 | PV441146 |
|  | 2 | *S. yunnanensis* | CLZhao 3647 | 99.74 | 1382 | 100 | 853 | PV441161 |
|  | 3 | *S. bambusicola* | CLZhao 31774 | 99.07 | 1354 | 100 | 1781 | PV197940 |
|  | 4 | Agaricales sp. | He 5464 | 98.81 | 1343 | 100 | 1823 | PQ842628 |
|  | 5 | Agaricales sp. | MD01 | 98.68 | 1338 | 100 | 1291 | MN515229 |
| nLSU  Dai 40350 | 1 | *S. bambusicola* | CLZhao 31774 | 97.56 | 3007 | 100 | 1781 | PV197940 |
|  | 2 | Agaricales sp. | He 5464 | 97.11 | 2961 | 100 | 1823 | PQ842628 |
|  | 3 | *S. bambusicola* | CLZhao 35394 | 97.21 | 2841 | 96 | 1682 | PV197941 |
|  | 4 | *S. punctata* | CLZhao 35563 | 97.28 | 2820 | 96 | 1686 | PV197942 |
|  | 5 | *M. aurantiidisca* | AFTOL-ID 1685 | 89.35 | 2191 | 100 | 1800 | DQ470811 |
| nLSU  Dai 40352 | 1 | Agaricales sp. | He 5464 | 97.43 | 3020 | 100 | 1823 | PQ842628 |
|  | 2 | *S. bambusicola* | CLZhao 31774 | 97.46 | 3005 | 99 | 1781 | PV197940 |
|  | 3 | *S. punctata* | CLZhao 35563 | 97.28 | 2857 | 95 | 1686 | PV197942 |
|  | 4 | *S. bambusicola* | CLZhao 35394 | 96.98 | 2822 | 95 | 1682 | PV231418 |
|  | 5 | *M. aurantiidisca* | AFTOL-ID 1685 | 89.09 | 2169 | 99 | 1800 | DQ470811 |
| nLSU  Dai 40355 | 1 | *S. bambusicola* | CLZhao 31774 | 97.45 | 3003 | 100 | 1781 | PV197940 |
|  | 2 | Agaricales sp. | He 5464 | 97.45 | 3001 | 100 | 1823 | PQ842628 |
|  | 3 | *S. punctata* | CLZhao 35563 | 97.28 | 2857 | 96 | 1686 | PV197942 |
|  | 4 | *S. bambusicola* | CLZhao 35394 | 96.98 | 2822 | 96 | 1682 | PV231418 |
|  | 5 | *M. aurantiidisca* | AFTOL-ID 1685 | 89.09 | 2169 | 100 | 1800 | DQ470811 |
| *TEF1*  Dai 40349 | 1 | *S. yunnanensis* | CLZhao 16450 | 96.62 | 782 | 100 | 633 | PV730093 |
|  | 2 | *S. yunnanensis* | CLZhao 3647 | 96.35 | 765 | 99 | 697 | PV730092 |
|  | 3 | *S. bambusicola* | CLZhao 31774 | 91.31 | 643 | 100 | 869 | PV231417 |
|  | 4 | *S. bambusicola* | CLZhao 35394 | 91.31 | 643 | 100 | 869 | PV231418 |
|  | 5 | *P. antillarum* | FJAU78352 | 84.10 | 449 | 100 | 1067 | PX857978 |
| *TEF1*  Dai 40350 | 1 | *S. yunnanensis* | CLZhao 16450 | 96.61 | 782 | 100 | 633 | PV730093 |
|  | 2 | *S. yunnanensis* | CLZhao 3647 | 96.35 | 765 | 99 | 697 | PV730092 |
|  | 3 | *S. bambusicola* | CLZhao 31774 | 91.31 | 643 | 100 | 869 | PV231417 |
|  | 4 | *S. bambusicola* | CLZhao 35394 | 91.31 | 643 | 100 | 869 | PV231418 |
|  | 5 | *P. fraxinophilus* | FJAU78347 | 83.92 | 449 | 100 | 661 | PX857982 |
| *TEF1*  Dai 40352 | 1 | *S. yunnanensis* | CLZhao 16450 | 94.74 | 621 | 85 | 633 | PV730093 |
|  | 2 | *S. yunnanensis* | CLZhao 3647 | 94.66 | 610 | 84 | 697 | PV730092 |
|  | 3 | *S. bambusicola* | CLZhao 31774 | 91.48 | 549 | 85 | 869 | PV231417 |
|  | 4 | *S. bambusicola* | CLZhao 35394 | 91.48 | 549 | 85 | 869 | PV231418 |
|  | 5 | *P. chlorocystis* | iNAT:217417973 | 83.84 | 431 | 97 | 477 | PX866552 |
| *TEF1*  Dai 40355 | 1 | *S. yunnanensis* | CLZhao 16450 | 94.71 | 736 | 87 | 633 | PV730093 |
|  | 2 | *S. yunnanensis* | CLZhao 3647 | 94.22 | 713 | 86 | 697 | PV730092 |
|  | 3 | *S. bambusicola* | CLZhao 31774 | 91.97 | 664 | 87 | 869 | PV231417 |
|  | 4 | *S. bambusicola* | CLZhao 35394 | 91.97 | 664 | 87 | 869 | PV231418 |
|  | 5 | *P. antillarum* | HMAS52750 | 83.43 | 475 | 94 | 517 | PP554378 |
